# The association between prenatal maternal selenium concentration and mental health in early childhood, a mother-child cohort study

**DOI:** 10.1371/journal.pone.0360527

**Published:** 2026-10-09

**Authors:** Suman Ranjitkar, Ingrid Kvestad, Ram K. Chandyo, Kjersti S. Bakken, Manjeswori Ulak, Tor A. Strand, Sandra Huber, Maria Averina, Mari Hysing

**Affiliations:** 1 Department of Psychosocial Science, Faculty of Psychology, University of Bergen, Bergen, Norway; 2 Department of Pediatrics, Child Health Research Project, Kathmandu, Nepal; 3 Women’s Clinic, Innlandet Hospital Trust, Lillehammer, Norway; 4 Community Medicine Department, Kathmandu Medical College, Kathmandu, Nepal; 5 Siddhi Memorial Hospital, Bhimsensthan, Bhaktapur, Nepal; 6 Center for International Health, University of Bergen, Bergen, Norway; 7 Department of Research, Innlandet Hospital Trust, Lillehammer, Norway; 8 Department of Laboratory Medicine, University Hospital of North Norway, Tromsø, Norway; 9 Department of Clinical Medicine, The Arctic University of Norway, Tromsø, Norway; King Faisal Specialist Hospital and Research Center, SAUDI ARABIA

## Abstract

**Background:**

Adequate selenium (Se) status is important for biological mechanisms involved in cognitive functioning and mental health. However, there are few studies that have examined the association between prenatal maternal Se and mental health of preschool children in low-and middle-income countries. The primary aim of this study was to measure associations between maternal plasma Se concentration during pregnancy and child mental health at four years. The secondary aim was to assess the psychometric properties of the parent reported Nepalese version of the Strengths and Difficulties Questionnaire (SDQ-P).

**Method:**

A total of 720 mother-infant dyads were included in the study from a cohort from Bhaktapur, Nepal, with prenatal maternal plasma Se concentration measured in early pregnancy. Child mental health was assessed at 4 years of age by the SDQ-P. The fit of the original five-factor structure of the SDQ-P was examined using Confirmatory Factor Analysis (CFA). Generalized linear models were used to analyze the association between maternal plasma Se concentration (continuous, dichotomized or tertiles) and child’s SDQ-P scores using the five-factor model.

**Results:**

The mean (SD) maternal plasma Se concentration was 74.7 µg/L (± 10.2 µg/L). The original five-factor structure of the SDQ-P was confirmed with some modifications (Comparative fit index (CFI)=0.967, Root mean square error of approximation (RMSEA)=0.059). Children with mothers with the lowest tertile of maternal plasma Se concentration had higher Hyperactivity-Inattention scores than those in the middle tertile (adjusted β = 0.329, 95% CI 0.023 to 0.636). No associations were observed between maternal plasma Se concentration and the other SDQ-P subscale scores.

**Conclusion:**

We observed limited evidence of an association between maternal plasma Se concentration and mental health outcomes in children at 4 years of age. The observed association between maternal plasma Se concentrations and Hyperactivity-Inattention was only present when comparing the lowest to middle tertile, however no clear exposure-response pattern was observed. This isolated finding requires confirmation in further studies.

## Introduction

Mental health problems are common among preschool children [1,2], and a high burden in low-and middle-income countries (LMIC) [3]. Risk factors of early mental health problems include poverty, lack of learning opportunities at home, inappropriate parenting, and poor nutrition [4,5]. Nutritional deficiency is a major burden in LMICs, including Nepal [6,7]. Previous studies indicate that a substantial proportion of pregnant women (35.6%) in Nepal have selenium deficiency [8], and mental health problems in children are frequent in Nepal [9,10].

Selenium (Se) is an essential micronutrient critical for several biological functions in human beings, including brain development and neurological function [11]. Se and Selenoproteins are responsible for neurotransmitter functioning [12], including the regulation of Gamma-aminobutyric acid and dopamine. Dysfunctions in these neurotransmitters are associated with several mental illnesses [13,14]. Previous studies have also demonstrated that Se plays a crucial role in reducing oxidative stress to protect DNA from damage and inflammation, which is a common feature of several mental disorders [15]. Se has also been associated with mental health in adults [16–18]. Some studies have reported an association between child Se concentration measured in sixth grade and anxiety in early adolescence (11–14 years old children) [19], as well as with Attention-Deficit /Hyperactivity Disorder (ADHD) [20–22] and autism spectrum disorder (ASD) [23].

A limited number of studies have examined the associations between prenatal Se and general mental health problems in children. In a US study, prenatal maternal Se concentration in the second and third trimester were inversely associated with mental health problems in middle childhood using the total difficulties score of the parent reported Strengths and Difficulties Questionnaire (SDQ-P) [24]. A Polish study also reported inverse associations between cord blood Se concentration and child mental health problems, but this association was limited to the emotional problems subscale of the SDQ [25]. Most studies on the association between Se and mental health in children are from high-income countries, and we have not identified any studies in preschool children. Given the high burden of nutritional deficiencies [26] and mental health problems [27] in LMICs, it is important to assess the link between nutritional status and mental health of preschool children in such settings.

Several factors may influence the association between maternal Se concentration and child mental health, including the sex of the child. A study from Denmark reported negative associations between a marker of maternal Se status, the activity of glutathione peroxidase 3 (GPx3) in the 3rd trimester, and ADHD symptoms in male children, but not in females [22]. However, a study from the USA, showed an association between maternal Se concentration using red blood cells and the risk of ADHD in females, but not males [20]. Socioeconomic factors and maternal age are associated with Se status in children [28,29], and are also linked to children’s mental health [30,31]. Due to their potential contribution, these factors need to be accounted for when investigating the relationship between Se concentration and the mental health of children.

Valid and reliable mental health assessments in a given setting require assessment tools demonstrating strong psychometric properties [32]. There are several reliable tools available to assess mental health of children [33]. The SDQ-P is a widely used brief tool to measure mental health problems in children from age two to 17 years [34]. Though there are authorized translated versions of SDQ-P in various languages including a Nepalese version [35–37], its psychometric properties in Nepal are, to our knowledge, still unknown. A review concluded that there are strong psychometric properties of SDQ across different populations aged four-12 years, largely supporting the original five-factor model (i.e., Emotional problems [EP], Hyperactivity-Inattention [HI], Conduct problems [CP], Peer problems [PP] and Prosocial behavior [PS]) [38]. The original five-factor structure was also found to be acceptable in Swedish children aged three to five years with good internal consistencies in all subscales [39]. However, a comparative study of the SDQ’s psychometric properties across different European settings was inconclusive, giving support to both the three-factor and five-factor models [40]. A low risk population based British study utilized the broader subscales of externalizing (HI and CP) and internalizing (EP and PP) problems instead of the specific subscales (EP, HI, CP and PP), showing better model fit than the original five-factor model [41]. In a LMIC setting, a study from India reported good fit for the five-factor model with the Root mean square error of approximation (RMSEA) value, but poor fit with the Comparative fit index (CFI) [42].

In a large mother-child cohort from Bhaktapur, Nepal, we have previously examined the associations between maternal plasma Se concentration and developmental outcomes during infancy [43] and in early childhood [8] showing no association. The present study differs in both outcomes and developmental period. It examines mental health, particularly emotional and behavioral difficulties, at 4 years of age (preschool age) in the same cohort using the SDQ-P. Although neurodevelopment and mental health are interrelated and together contribute to children’s overall development, they represent distinct developmental domains and thus may have differential associations with prenatal maternal plasma Se concentration.

The primary aim of the current study was to explore the association between maternal plasma Se concentration and SDQ-P scores. Since the psychometric properties of the SDQ-P have not been previously examined in a Nepalese setting, a secondary aim was to measure the model fit of the SDQ-P in this setting.

## Methods

### Study design and setting

The participating mothers and children were enrolled during pregnancy and followed up until the child was four years of age. The mothers were originally enrolled in a study entitled “Supplementation of vitamin B_12_ in pregnancy and postpartum on growth and neurodevelopment in early childhood: A Randomized, Placebo Controlled Trial” [44]. In total, 800 pregnant women were randomly assigned to daily supplementation of 50 microgram of vitamin B_12_ or a placebo (1:1 ratio) from the day of enrollment to 6 months postpartum. We included all mother-infant dyads with maternal plasma Se concentration during early pregnancy and SDQ-P data at 4 years follow-up (N = 720), constituting 95% of the total live births (N = 760).

The study was conducted in a semi-urban setting of Bhaktapur municipality and its surrounding areas. Bhaktapur is the neighboring district of the capital city of Kathmandu, Nepal, and one of the most densely populated areas in the country. In the census of 2021, the total population of Bhaktapur was 432,132. The major professions in this setting are agriculture, small-scale business, services at government and private sectors and daily wage-based jobs. Most of the children are sent to child- or daycare centers before 2 years of age, while such centers are targeted for 3–5-year-olds.

### Procedure

From 27 March 2017–16 October 2020, pregnant women within 15 weeks of gestation were enrolled. Inclusion criteria were within 15 weeks of gestation, age 20–40 years, and planning to stay in Bhaktapur for the next two years. Exclusion criteria were taking multivitamins including vitamin B_12_, having chronic illnesses, severe anemia, and having a body mass index (BMI) outside the range of 18.5–29.9 kg/m^2^. Participants were followed up twice a week until 6 months postpartum to collect information about the compliance of the supplementation, hospital visits and morbidity. The study procedures are presented in Fig 1.

**Fig 1:**
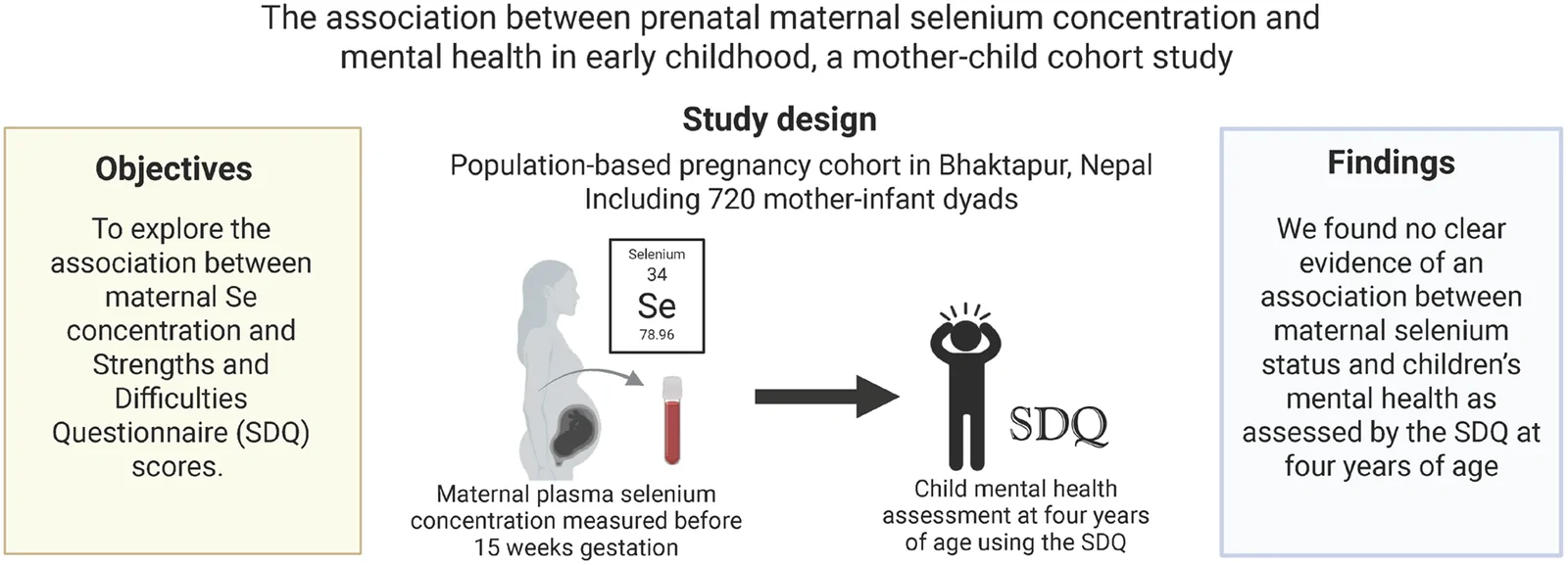
Study design for major activities at the enrolment and at the age of 48 months in Nepalese mothers and children.

### Sociodemographic and clinical information

All sociodemographic, clinical and biological information was collected at enrollment. Parental level of education was collected as the number of years of completed schooling at enrollment and categorized as below or above secondary school. Parental occupation was collected as specific titles (housewife, carpet worker, daily wage earner, agriculture, business, services in the private sector, government employee, and foreign employment) and the response category housewife was regarded as no formal work and the rest as formal work. Caste of the participants were categorized as Newar, Brahmin/Chhetri, Indigenous groups (Gurung/Rai/Magar/Tamang/Lama), and others. Women’s height and weight were measured at enrollment, and their BMI was calculated as kilogram per square meter (kg/m^2^). We categorized family types as nuclear or joint, housing status as own or rented house and land ownership and whether the family received remittance from abroad as yes or no. We have also collected information about the smoking habits of the pregnant women, but since few reported to be smoking regularly (n = 2) and previously (n = 1), we excluded smoking from the analysis although it could be a potential confounder [45,46].

The socioeconomic status of the participants was estimated based on the WAMI index [47]. This index is calculated based on variables on Water and sanitation, Assets ownership, Maternal education, and household Income reported by the parents at enrollment. Details of the WAMI index calculation is published elsewhere [8,47].

After delivery, we measured the child’s weight and length. Gestational age was collected as weeks of gestation based on the last menstrual period of the pregnant mother, further confirmed through USG report, and being born <37 weeks of gestation was considered as preterm birth. Low birth weight (LBW) was defined as <2500 g.

### Lab procedure

Venous blood samples (3–4 mL) were obtained from the pregnant women at enrollment by experienced, well-trained laboratory technicians. Samples were centrifuged at 2000 x g for 10 minutes, after which plasma was separated in cryotubes, stored at −70°C in a local laboratory, and shipped to Norway on dry ice.

### Selenium concentration

All the samples were analyzed in the Environment Pollutant Laboratory, at the University Hospital of North Norway, Tromsø, Norway. Plasma samples were diluted with alkaline reagent (1:20) by a liquid handler. In kinetic energy discrimination mode with the flow of helium gas at 4.8 mL, instrumental analysis was performed by inductively coupled plasma mass spectrometry (Nexion 300D, Perkin Elmer, Waltham, MA, USA). Rhodium (Inorganic Ventures, Christiansburg, VA, USA) acted as internal standard. Matrix matched calibration curves were prepared with ClinCal plasma samples (Recipe Chemicals and Instruments GmbH, Germany). Two sets of control samples were analyzed with each batch of 32 samples from Recipe Chemicals, Germany and SeroAS, Norway, (ClinChek plasma and Seronorm serum, both level 1 and 2) and their coefficients of variations were 5% and 6% respectively. The method detection limit for Se was set as 2.39 µg/L based on the standard deviation detected out of three times in 105 blank sample of Se concentration. All laboratory procedures were validated, and analyses were performed by trained and qualified laboratory technicians in accordance with the clinical accreditation standard NS-EN IS 15189:2022. For the general population, plasma Se concentration of 80–95 μg/L is considered the optimum level for enzyme functioning [48]. For the present work, we used Norwegian maternal Se concentration of 71.1 μg/L as a cut-off to indicate deficiency [49]. The percentile distribution of the sample has been published elsewhere [8].

### Mental health assessment

The SDQ is a widely used mental health assessment tool for children, developed in the United Kingdom (UK) with norms based on UK samples [34]. The SDQ is a standardized tool with different versions; in the current study we used the parent-reported version with an authorized Nepalese translation obtained from the developer (sdqinfo.org). The SDQ-P consists of 25 main items along with additional follow-up items. The response options for the main items are ‘*Not true*’, ‘*Somewhat true*’ and ‘*Certainly true*’. It has five original subscales: Emotional problems (EP), Hyperactivity-Inattention (HI), Conduct problems (CP), Peer problems (PP) and Prosocial behaviors (PS). Scores from the four problem subscales are summed up to derive the total difficulties score. Higher scores indicate greater mental health problems in children. For the PS subscale, higher scores indicate better functioning. For the current study, the questionnaire was administered to caregivers by experienced and well-trained field workers at the field site, in a room free from distractions when the child was four years of age. Regular refresher training was provided by a supervising psychologist to ensure high data quality.

### Statistical analysis

All basic demographics and clinical information are presented as numbers (N) and percentages (%) if the variables were categorical or means (M) and standard deviations (SDs) if the variables were continuous. Missing data is handled using listwise deletion.

The 25 SDQ-P items were submitted to a confirmatory factor analysis (CFA) with the Weighted Least Squares Mean and Variance (WLSMV) estimator since data were ordinal and skewed, using the JASP program (**version 0.95.4**). The original five-factor model consists of the five subscales: EP, HI, CP, PP and PS (**Model 1**), while the bi-factor model consists of Externalizing problems, Internalizing problems and Prosocial behavior subscales (**Model 2**).

Model fit was estimated based on the CFI and the RMSEA, where CFI values greater than 0.95 and RMSEA less than 0.06 was considered good model fit and CFI values less than 0.95 and greater than 0.90 and RMSEA values greater than 0.05 was considered acceptable fit [50,51]. Based on the modification indices, the largest and conceptually matched items were freed from the tandem, and the model was re-run. This process continued until we found an acceptable model fit. Internal consistency of the scale was measured using Omega (ω), and values  > 0.7, were considered acceptable [52].

Before conducting the main analyses, we assessed the appropriateness of the planned statistical models using model diagnostics, including histogram, fractional polynomial tests, Q-Q plots, residual vs. fitted plots and hyperbolic associations using quadratic terms. Homoscedasticity, linearity, and normality of residuals were considered acceptable, and we therefore used generalized linear models (GLMs) with a Gaussian family and identity link function to examine associations between maternal plasma Se concentration (μg/L) and SDQ-P scores (based on the CFA result) using Stata (V19.5). We also examined the associations between maternal plasma Se concentrations categorized into tertiles and dichotomized using the cut-off based on a Norwegian pregnant population (<71.1 μg/L) [49] and SDQ-P subscale scores. We also included child sex and maternal plasma Se concentration as an interaction term in the regression models. We used robust standard errors in all models. Details of the plan of analysis can be found at the preregistration registered on 18.08.2025 [53].

### Ethics

Our study obtained ethical approval from the Nepal Health Research Council (NHRC; 253/2016, follow-up approval 423/2023) and from the Regional Committee for Medical and Health Research Ethics in Norway (REK vest; reference number 2016/1620). Written informed consent from the mothers was obtained after providing thorough information on the study activities and additional consent was obtained at three years of age for further follow-up until five years of age.

## Results

### Sociodemographic and nutritional characteristics

The mean age (SD) of the pregnant women at enrollment was 27.6 (3.9) years. Of the participants, 66.9% were engaged in formal work, and 57.1% had completed secondary level education. Overall, 69.2% of the families owned land, 23.6% lived in rented housing, and 26.2% were living in a single room serving as both kitchen and bedroom. Among the children, 52.6% were male, 7.9% born preterm, and 12.6% had a birth weight of less than 2500 g (**Table 1**).

**Table 1:** Sociodemographic and clinical information of 720 mothers from Bhaktapur, Nepal.

Demographic and socioeconomic features	N = 720
Mother’s age, mean **±** SD	27.6 (3.9)
Mothers who completed secondary school or above	412 (57.1%)
Mothers who work	482 (66.9%)
**Caste/Ethnicity**	
Newar	572 (79.5%)
Brahmin /Chhetri	68 (9.4%)
Indigenous group	61 (8.5%)
Others	19 (2.6%)
**Socio-economic status**	
Monthly income ^¶^ High (above 75k)	60 (8.3%)
Medium (above 25k–75k)	381 (53.0%)
Low (Up to 25k)	279 (38.7%)
Family staying in joint family	474 (65.8%)
Family residing in rented house	170 (23.6%)
Kitchen and bedroom in same room	189 (26.2%)
Family having own land	498 (69.2%)
Receiving remittance from abroad	76 (10.6%)
WAMI^ֆ^ **(n = 716)**	0.66 (0.13)
**Nutritional status of mother**	
Weight (kg), mean (SD)	55.2 (7.5)
Maternal BMI (kg/m^2^), mean (SD)	23.7 (3.0)
**Micronutrient status of mother**	
Maternal plasma Selenium concentration (µg/L), mean (SD)	74.7 (10.2)
Low (<71.1 µg/L)	258 (35.8%)
Normal (≥71.1 µg/L)	462 (64.2%)
Lowest Tertile (n = 237)	63.9 (4.7)
Middle Tertile (n = 244)	74.4 (2.3)
Highest Tertile (n = 239)	86.1 (7.2)
	
**Child characteristics**	
Male	379 (52.6%)
Gestational week, mean (SD)	38.5 (1.6)
Preterm birth	57 (7.9%)
Low birth weight	91 (12.6%)

¶ Nepalese Rupees. ^ֆ^ Water and sanitation, Assets ownership, Maternal education, and household Income.

All figures are n(%) unless otherwise specified.

### Confirmatory factor analysis of SDQ-P

The CFA (**Model 1**) on the original five-factor structure did not converge, with estimation problems associated with the item *Attends.* Inspections of the correlation matrix showed weak associations with most of the other items in the Hyperactivity-Inattention (HI) subscale, except for the *Reflect* item. After removing the item *Attends,* the model converged but showed poor fit (CFI = 0.858, RMSEA = 0.125). The inspection of the modification indices revealed potential cross-loadings; the item *Reflect*: reverse coded (from HI subscale) also loaded on the Prosocial behavior (PS) subscale (not included in PS subscale for analyses) and *Obeys*: reverse coded item (from Conduct problems (CP) subscale) also cross-loaded on HI subscale. Allowing both items to cross load and constraining *Reflect* item to be positive in the HI subscale, the model was not approved in an acceptable range. Further inspecting the model results, we found that the item *Obeys* behaved as a Heywood case with factor loadings in CP (−5.019) and HI (5.098). We therefore removed this item from the model. The factor loading of the item *Bullied* also seems problematic, with low loading and weak correlations with other items of the same subscale. We therefore decided to exclude this item from the model as well. After adjustment with some modification index (*Worries* and *Loner* (r = 0.622); and *Restless* and *Fidgety* (r = 0.262), the model improved to a good fit (CFI = 0.967, RMSEA = 0.059) (**Table 2**). All the subscales were derived excluding problematic items (i.e., *Obeys* from the CP subscale*, Attends* from the HI subscale and *Bullied* from the PP subscale) based on the final model. All original items were retained for the EP and PS subscales.

**Table 2:** Model fit indices for the confirmatory factor analysis of the SDQ-P structure with and without modifications (n = 720).

Models	CFI	RMSEA (CI)
**Model 1:** Five-factor model (Excluding item: *Attends*)	0.858	0.125 (0.121, 0.129)
**Model 1:** Five-factor model with modification ^Ϯ^	0.967	0.059 (0.054, 0.064)
**Model 2:** Bi-factor model (Excluding item: *Attends*)	0.738	0.137 (0.132, 0.142)
**Model 2:** Bi-factor model with modification ^ϮϮ^	0.959	0.054 (0.048, 0.060)

CFI, Comparative Fit Index; RMSEA, Root mean square error of approximation; CI, Confidence Interval

^Ϯ^Excluding *Attends*, Cross-loadings *Reflect* on Prosocial, excluding *Obeys* and *Bullied* from the model, constraining *Reflect* to be positive and correlating to error items *Worries* with *Loner* and *Restless* with *Fidgety.*

^ϮϮ^Correlating Externalizing and Internalizing problems, cross-loading *Obeys* on Internalizing problems, excluding *Obeys* item from Externalizing problems, excluding items *Somatic* and *Loner* from Internalizing problems and constraining the item *Oldbest* to be positive on Internalizing problems.

In **model 2**, we used a bifactor model of SDQ-P with Externalizing problems, Internalizing problems and Prosocial behavior. The model did not converge, but we could run the model excluding the item *Attends* with CFI = 0.738, RMSEA = 0.137. Modification indices showed that the Externalizing problems and Internalizing problems were correlated. When we allowed them to correlate in the model, the model fit indices were good with CFI = 0.975 and RMSEA = 0.042, however, the factor loadings were not promising. After modification indices, we concluded a final good model fit with CFI = 0.959 and RMSEA = 0.054 (**Table 2**). We decided not to use this model in the final analysis due to the need to correlate Externalizing problems with Internalizing problems to obtain a good fit, which is theoretically against the principle of bi-factors model in CFA [54].

The median scores, IQR and range of Emotional problems (EP), Hyperactivity-Inattention (HI), Conduct problems (CP), Peer problems (PP) and Prosocial behaviors (PS) are presented in **Table 3**. Internal consistency (ω) of all subscales ranged from 0.47 to 0.79, except for the PP subscale with a substantially poor score (ω = 0.05). The coefficient of PS is in an acceptable range, whereas the rest of the subscales are in a questionable range.

**Table 3:** Distribution and reliability of SDQ-P subscales scores of preschool children in Bhaktapur, Nepal (N = 720).

Variables^¤^	Median (IQR)	Range	Omega
Emotional problems	3 (1,4)	0-10	0.65
Conduct problem	2 (1,4)	0-8	0.47
Hyperactivity^a^	3 (2,4)	0-8	0.49^b^
Peer problems	3 (2,4)	0-7	0.05
Prosocial behavior	8 (5,10)	0-10	0.79

^a^*With all items in the model (omega = 0.47),* ^b^ Excluding *Attends.*

### Association between maternal plasma Se concentration and SDQ-P subscale scores

**Tables 4** and **5** show the associations between maternal plasma Se concentration and SDQ-P subscale scores based on the five-factor model (**Model 1**). As per the original plan of analyses, interaction terms were included in each model, but the interactions were not significant, hence the associations are examined separately. The results from all models for interaction terms are presented in S1 Table. There were no associations between maternal plasma Se concentration and SDQ-P subscale scores (continuous and dichotomized) without and with adjustment for confounders (**Table 4**). Analyzing the maternal plasma Se concentration as tertiles, children of mothers in the lowest tertile had higher Hyperactivity-Inattention scores than those whose mothers were in the middle tertile. This association was statistically significant in both the crude model (Coeff. 0.316 (95% CI: 0.010, 0.623) and the adjusted model (Coeff. 0.329 (95% CI: 0.023, 0.636) (**Table 5**). The other subscales did not show any association with the maternal plasma Se concentrations.

**Table 4:** Association between maternal plasma Selenium concentration and the Strengths and Difficulties Questionnaire scores of children of Bhaktapur, Nepal using regression analysis (N = 720).

			Crude			Adjusted^֎^	
**SDQ subscale scores**	**Se exposure**	**Coeff.**	**CI**	**p-value**	**Coeff.**	**CI**	**p-value**
Emotional problems	Continuous	−0.006	−0.022, 0.011	0.493	0.004	−0.021, 0.012	0.609
	Deficient^c^	Ref.					
	Normal	0.219	−0.127, 0.564	0.214	0.208	−0.136, 0.552	0.236
Conduct problems	Continuous	−0.004	−0.020, 0.008	0.519	−0.004	−0.016, 0.009	0.575
	Deficient	Ref.					
	Normal	0.065	−0.204, 0.334	0.636	0.071	−0.199, 0.341	0.605
Hyperactivity	Continuous	−0.005	−0.018, 0.007	0.401	−0.005	−0.018, 0.008	0.448
	Deficient	Ref.					
	Normal	0.181	−0.087, 0.449	0.187	0.182	−0.086, 0.450	0.184
Peer problems	Continuous	−0.000	−0.011, 0.009	0.934	0.0004	−0.010, 0.011	0.934
	Deficient	Ref.					
	Normal	0.110	−0.100, 0.319	0.304	0.092	−0.116, 0.030	0.386
Prosocial behaviors	Continuous	−0.001	−0.019, 0.016	0.871	−0.001	−0.019, 0.016	0.889
	Deficient	Ref.					
	Normal	0.030	−0.311, 0.371	0.863	0.022	−0.318, 0.362	0.898

^c^Maternal plasma Selenium concentration <71.1μg/L. Adjusted for maternal age, WAMI, and Sex of the child, Reference group for sex of the child: Male, ^**֎**^ n = 716.

**Table 5:** Associations between maternal plasma selenium concentration (Tertiles) and SDQ-P scores of children in Bhaktapur Nepal at 48 months using regression analysis (N = 720).

	Crude			Adjusted^Ϯ^		
SDQ subscale scores Se concentration	Coeff.	95% CI	p-value	Coeff.	95% CI	p-value
Emotional problems						
Lowest Tertile	0.029	−0.368, 0.425	0.887	0.020	−0.376, 0.416	0.921
Middle Tertileª	Ref.			Ref.		
Highest Tertile	−0.293	−0.685, 0.098	0.142	−0.280	−0.674, 0.113	0.163
Conduct problems						
Lowest Tertile	0.145	−0.168, 0.459	0.363	0.162	−0.152, 0.476	0.312
Middle Tertileª	Ref.			Ref.		
Highest Tertile	0.120	−0.191, 0.429	0.452	0.142	−0.167, 0.451	0.369
Hyperactivity						
Lowest Tertile	0.316	0.010, 0.623	0.043	0.329	0.023, 0.636	0.035
Middle Tertileª	Ref.			Ref.		
Highest Tertile	0.133	−0.185, 0.453	0.411	0.151	−0.170, 0.472	0.357
Peer problems						
Lowest Tertile	0.080	−0.163, 0.321	0.523	0.070	−0.175, 0.308	0.587
Middle Tertileª	Ref.			Ref.		
Highest Tertile	0.047	−0.279, 0.186	0.693	−0.034	−0.268, 0.199	0.775
Prosocial behaviors						
Lowest Tertile	0.035	−0.360, 0.428	0.862	0.004	−0.392, 0.399	0.986
Middle Tertileª	Ref.			Ref.		
Highest Tertile	0.070	−0.344, 0.483	0.741	0.040	−0.376, 0.448	0.683

Adjusted for maternal age, WAMI, and Sex of the child, ª Reference group, Reference group for sex of the child: Male, ^**Ϯ**^ n = 716.

## Discussion

In a sample of 720 Nepalese children, there were no associations between maternal plasma Se concentration and the SDQ-P subscale scores except for Hyperactivity-Inattention subscale. To our knowledge, the present work is the first study to investigate the association between maternal plasma Se concentration and general mental health of preschool children in a LMIC.

Overall, our findings provide limited evidence of an association between prenatal maternal plasma Se concentration and mental health in these Nepalese preschool children. Our findings is in contrast with a US study that reported associations between prenatal maternal Se concentration and child mental health measured by the SDQ total score [24]. However, SDQ was measured in middle childhood, and thus developmental differences in early- and middle-childhood could account for the contrasting outcomes. In addition, contrasting to our study, a Polish study identified an association between cord blood Se and the emotional problems subscale of the SDQ [25]. It is possible that there are better methods to measure Se status, such as full-blood Se concentration, or enzymatic activity [55,56]. The use of different biomarkers, tissues and timing of sample collection may explain the heterogeneity between the different study findings.

Notably, our findings provide some evidence that lower maternal plasma Se concentration is associated with Hyperactivity-Inattention in preschool children. Children with maternal plasma Se concentration in the lowest tertile had higher Hyperactivity-Inattention scores compared with those with Se concentration in the middle tertile. No such association was observed for children with maternal plasma Se concentration in the highest tertile, and no associations were found using Se on a continuous scale or dichotomized. These association was in line with a findings from a previous study where there was an inverse association between maternal Se concentration during pregnancy and ADHD trait at 5 years of age [22], but not with results from a US study reporting positive association between higher maternal Se concentration and clinically diagnosed ADHD [20].

To ensure that we had a reliable outcome measure, we performed CFA of two widely used models of the SDQ-P, and a modified five-factor solution was supported. This included removing the single item *Attends* that showed weak associations with the other items in the Hyperactivity-Inattention (HI) subscale. The weak associations may be due to a slight back-translation discrepancy of the item *Attends* or it could be due to attention being less developed and harder for the parents to assess in four-year-old children [57,58]. We also did some cross-loadings, for example the reverse coded *Reflect* item from Hyperactivity-Inattention (HI) to Prosocial behavior (PS), which is theoretically meaningful given the overlap between these constructs [59] and this has also been done in previous studies [60] as cross-loadings of items can improve model fit [61]. Similarly, correlating the error terms between the items *Worries* with *Loner* to improve model fit are theoretically meaningful given the known association between social anxiety and loneliness [62–64]. Further correlating the error term (*restless* with *fidgety*) is a common finding in the literature on CFA of SDQ [65,66]. After these modifications the five-factor model showed good fit and was chosen as an outcome measure in further analysis.

Notably, the mental health problems based on the median scores of the SDQ-P subscales are at a similar level compared to previous studies [67,68], however, the level of emotional problems (EP) was higher in the present study. A similar pattern was observed in a previous study from Nepal, where the findings were attributed to disadvantaged family backgrounds and sociocultural factors affecting the reporting of internalizing problems, such as emotional problems in LMICs [10].

A key strength of our study is its relatively large sample size of 720 participants. All the laboratory procedures were conducted under excellent standards by trained and experienced laboratory technicians, and blood samples were stored at the field site and shipped to Norway following standard biobanking procedures [44]. We used a standardized and validated tool to measure mental health and conducted CFA to evaluate its psychometric properties. A strength is also the use of Se concentration on both a continuous scale and categorized based on known cut-offs and in tertiles to ensure that we did not overlook any meaningful associations with the SDQ-P score.

This study has some limitations. Given that hair and toenails are considered better options for long-term Se status [55], using plasma to measure Se concentration may not be an ideal matrix for estimating Se status [69], potentially leading to random misclassification. Random misclassification will increase the noise and thus also increase the risk of type 2 errors. Used as an exposure, this will also tend to attenuate the regression coefficients and accordingly underestimate the strength of the associations [70]. The findings observed in this study should also be interpreted cautiously in light of the psychometric characteristics of the SDQ-P in the current population. Although the five-factor model showed acceptable model fit, the original five-factor structure required modifications. Moreover, internal consistency is low for some subscales which may have reduced the precision of the measured constructs and potentially weakened the associations with maternal plasma Se concentration. Mental health measurement was based on questionnaire rather than clinical diagnostic interview and does not indicate the presence of psychiatric disorders. Additionally, the application of exclusion criteria to select a healthy population may limit the generalizability of the findings.

## Conclusion

In our cohort of healthy pregnant women, maternal plasma Se concentration in early pregnancy was not associated with child mental health, as measured by the SDQ-P at four years of age, except for the Hyperactivity-Inattention subscale. This latter observation may suggest that Se deficiency could be related to mental health, however, further studies are needed to confirm this finding.

## Supporting information

S1 Table Estimates of interaction between maternal plasma selenium concentration and child sex for SDQ outcomes (Interaction terms from the different models). (DOCX)

## Abbreviations

CFI: Comparative fit index
CP: Conduct problems
EP: Emotional problems
GLM: Generalized linear model
HI: Hyperactivity-Inattention
LMICs: Low- and middle-income countries
NHRC: Nepal Health Research Council
PP: Peer problems
PS: Prosocial behavior
RMSEA: Root mean square error of approximation
SD: Standard Deviation
SDQ-P: Parent reported Strengths and Difficulties Questionnaire
Se: Selenium
WAMI: Water and sanitation, Assets ownership, Maternal education, and household Income
