# Supplementary material for: The association between prenatal maternal selenium concentration and mental health in early childhood, a mother-child cohort study
Source: PLoS One. 2026 Oct 9;21(10):e0360527. doi: 10.1371/journal.pone.0360527 (PMC13652251; doi:10.1371/journal.pone.0360527)
Supplement: Supplementary file 1 [file pone.0360527.s001.docx]

**S1 Table: Estimates of interaction between maternal plasma selenium concentration and child sex for SDQ-P outcomes (Interaction terms from the different models)**

|  |  |  | **Adjusted** ^╚^ |  |
| --- | --- | --- | --- | --- |
| **SDQ subscale scores** | **Se exposure** | **Coeff.** | **CI** | **p-value** |
| Emotional problems | Continuous | -0.018 | -0.051, 0.015 | 0.291 |
|  | Deficient^c^ | Ref. |  |  |
|  | Normal | 0.482 | -0.207, 1.171 | 0.170 |
|  | Lowest Tertile | 0.263 | -0.533, 1.060 | 0.517 |
|  | Middle Tertileª | Ref. |  |  |
|  | Highest Tertile | -0.393 | -1.171, 0.386 | 0.323 |
| Conduct problems | Continuous | -0.010 | -0.034, 0.015 | 0.456 |
|  | Deficient | Ref. |  |  |
|  | Normal | 0.144 | -0.398, 0.686 | 0.602 |
|  | Lowest Tertile | 0.024 | -0.610, 0.655 | 0.942 |
|  | Middle Tertileª | Ref. |  |  |
|  | Highest Tertile | -0.380 | -0.992, 0.241 | 0.232 |
| Hyperactivity | Continuous | -0.013 | -0.040, 0.013 | 0.320 |
|  | Deficient | Ref. |  |  |
|  | Normal | 0.033 | -0.505, 0.571 | 0.904 |
|  | Lowest Tertile | -0.168 | -0.784, 0.450 | 0.593 |
|  | Middle Tertileª | Ref. |  |  |
|  | Highest Tertile | -0.436 | -1.072, 0.200 | 0.179 |
| Peer problems | Continuous | -0.016 | -0.036, 0.005 | 0.128 |
|  | Deficient | Ref. |  |  |
|  | Normal | 0.278 | -0.138, 0.694 | 0.190 |
|  | Lowest Tertile | 0.294 | -0.190, 0.780 | 0.233 |
|  | Middle Tertileª | Ref. |  |  |
|  | Highest Tertile | 0.023 | -0.442, 0.490 | 0.924 |
| Prosocial behaviors | Continuous | 0.014 | -0.021, 0.050 | 0.434 |
|  | Deficient | Ref. |  |  |
|  | Normal | -0.019 | -0.699, 0.661 | 0.956 |
|  | Lowest Tertile | 0.435 | -0.360, 1.230 | 0.283 |
|  | Middle Tertileª | Ref. |  |  |
|  | Highest Tertile | 0.720 | -0.103, 1.542 | 0.087 |

Adjusted for maternal age and WAMI, Interaction terms used (Selenium and Sex of the child), ª Reference group, Reference group for sex of the child: Male,^╚^ n=716
